# Hydrothermal Synthesis of Nickel Oxide and Its Application in the Additive Manufacturing of Planar Nanostructures

**DOI:** 10.3390/molecules28062515

**Published:** 2023-03-09

**Authors:** Darya A. Dudorova, Tatiana L. Simonenko, Nikolay P. Simonenko, Philipp Yu. Gorobtsov, Ivan A. Volkov, Elizaveta P. Simonenko, Nikolay T. Kuznetsov

**Affiliations:** 1 Chemistry of Light Elements and Clusters Laboratory, Kurnakov Institute of General and Inorganic Chemistry of the Russian Academy of Sciences, 119991 Moscow, Russia; 2 Institute of Materials for Modern Energy and Nanotechnology IMSEN-IFH, Mendeleev University of Chemical Technology of Russia, 125047 Moscow, Russia; 3 Sensor Microsystems Laboratory, Moscow Institute of Physics and Technology, National Research University, 141701 Dolgoprudny, Moscow Region, Russia

**Keywords:** hydrothermal synthesis, NiO, SDC, hierarchical structures, functional inks, nanosheets, nanospheres, microextrusion printing, electrode, SOFC

## Abstract

The hydrothermal synthesis of nickel oxide in the presence of triethanolamine was studied. Furthermore, the relationship between the synthesis conditions, thermal behavior, crystal structure features, phase composition and microstructure of semi-products, and the target oxide nanopowders was established. The thermal behavior of the semi-products was studied using a simultaneous thermal analysis (in particular, using one that involved thermogravimetric analysis and differential scanning calorimetry, TGA/DSC). An X-ray diffraction (XRD) analysis revealed that varying the triethanolamine and nickel chloride concentration in the reaction system can govern the formation of α- and β-Ni(OH)_2_-based semi-products that contain Ni(HCO_3_)_2_ or Ni_2_(CO_3_)(OH)_2_ as additional components. The set of functional groups in the powders was determined using a Fourier-transform infrared (FTIR) spectroscopy analysis. Using microextrusion printing, a composite NiO—(CeO_2_)_0.80_(Sm_2_O_3_)_0.20_ anode film was fabricated. Using XRD, scanning electron microscopy (SEM), and atomic force microscopy (AFM) analyses, it was demonstrated that the crystal structure, dispersity, and microstructure character of the obtained material correspond to the initial nanopowders. Using Kelvin probe force microscopy (KPFM) and scanning capacitance microscopy (SCM), the local electrophysical properties of the printed composite film were examined. The value of its conductivity was evaluated using the four-probe method on a direct current in the temperature range of 300–650 °C. The activation energy for the 500–650 °C region, which is of most interest in the context of intermediate-temperature SOFCs working temperatures, has been estimated.

## 1. Introduction

Today, it is becoming more evident that meeting the annually growing world demand for electricity is only possible through innovative development using the achievements of fundamental science and through the search and development of new materials, devices, and technologies of alternative energy [1,2,3,4]. In this context, solid oxide fuel cells (SOFCs) are of particular interest as one of the most promising electrochemical energy generators; they are capable of directly converting the energy of a chemical reaction into electrical energy [5]. Moreover, not only are they capable of using pure hydrogen as a fuel, but they are also capable of using various hydrocarbons (in particular, natural gas, methane, propane, and butane), bioethanol, and prepared diesel fuel [6,7,8]. An additional important advantage of SOFCs can be the considered heat generation (often in the form of water steam heated up to 150–250 °C) during their operation, which allows for a further increase in the efficiency of these devices (up to 80%) in their integration with gas and steam turbines [9,10,11].

The anode is one of the most important components for fuel cell operation, and it is the surface on which the electrocatalytic oxidation of fuel takes place. An effective SOFC anode material should have high ionic and electronic conductivity, catalytic activity, and a developed pore system that contributes to an increase in the number of active centers involved in the hydrogen oxidation reaction [12,13]. Moreover, the development of anode materials must take into account the compatibility of the materials’ linear thermal expansion coefficient values with these parameters for other functional layers of the fuel cell in order to ensure its correct operation in the temperature range of 500–800 °C [14]. Quite popular and commonly used SOFC anode materials are composites based on nickel or its oxide with the addition of a solid electrolyte based on cerium or zirconium dioxide, which is doped with yttrium, scandium, or other rare earth elements, e.g., Ni—GDC (Ni—CeO_2_-Gd_2_O_3_), NiO—SDC (NiO—CeO_2_-Sm_2_O_3_), NiO—YSZ (NiO—ZrO_2_-Y_2_O_3_), and Ni—ScSZ (Ni—ZrO_2_-Sc_2_O_3_) [15,16,17,18]. The anode materials based on nickel oxide and samarium-doped ceria (NiO—SDC) have a higher ionic conductivity in the intermediate-temperature range compared with the traditionally used yttrium-stabilized zirconia cermets and also demonstrate a higher chemical resistance to carbonization when using methane and other hydrocarbons as a fuel, which attracts particular attention in this context [19,20,21].

The functional characteristics of fuel cell anodes are significantly influenced by their microstructure, which can be controlled at the stage of synthesis of the corresponding materials. Recently, the attention of researchers in this field has been attracted by the methods of forming anisotropic nanomaterials with a hierarchical organization of the microstructure and a developed surface which, in turn, accelerates the charge transfer processes and enables a significant improvement of the corresponding materials’ electrophysical properties [22,23,24,25,26,27]. According to data from the literature, one of the most convenient methods for obtaining self-organized nanomaterials is by using the hydrothermal method which, depending on the process conditions, allows for the formation of solid phase particles of different shapes and dispersions and is characterized by the necessary crystal structure type and crystallinity degree [28,29,30,31]. In the majority of works devoted to the hydrothermal synthesis of nickel hydroxide or nickel oxide, the base medium is provided by sodium hydroxide, ammonia hydrate, and urea [32,33,34,35,36,37]. Triethanolamine, which can additionally play the role of a complex- and structure-forming agent, was only used as a base in single works; however, this process is still poorly studied [38,39].

A further increase in the performance of SOFCs is not only possible by using intermediate-temperature functional layers but also by moving towards planar-type cells. To date, a wide range of methods are used to implement this problem: screen printing [40,41,42], tape casting [43,44], the infiltration method [45,46], spray pyrolysis [47,48], and sputtering methods [49,50]. Nevertheless, these approaches do not always allow for the achievement of the required lateral resolution, reproducibility of microstructural features, and desired area and geometry of the planar structures. Printing technologies are an alternative to the above methods, which can considerably reduce the above limitations. Today, methods such as inkjet printing [51,52,53,54], aerosol printing [55,56,57,58], microplotter printing [59,60,61], microextrusion printing [62,63,64] and pen plotter printing [65,66] allow for the use of different types of inks (both true solutions and powder-based disperse systems) to form coatings of desired thickness and geometry on substrates of different natures. The above approaches are being actively developed in the context of obtaining materials for alternative energy, electronics, gas sensing, and optics.

This work aimed to study the hydrothermal synthesis of nickel oxide in the presence of triethanolamine, establish the dependences between the reagent concentration, crystal structure features, phase composition, and microstructure of intermediate and target products, and develop an approach for the microextrusion printing of NiO-SDC films, which are promising as anode functional layers.

## 2. Results and Discussion

### 2.1. Examination of Semi-Products and NiO Nanopowders

In this work, in order to establish the influence of reagent concentrations and their quantitative ratio on the semi-products and final products properties, the specified parameters were varied in a sufficiently wide range (Table 1). Thus, the nickel chloride concentration ranged from 0.005 to 0.100 mol/L and triethanolamine concentration ranged between 0.010 and 0.400 mol/L. Thus, the reagent ratios (c(TEA)/c(Ni^2+^)) were changed in the range of 2–80.

**Figure 1:**
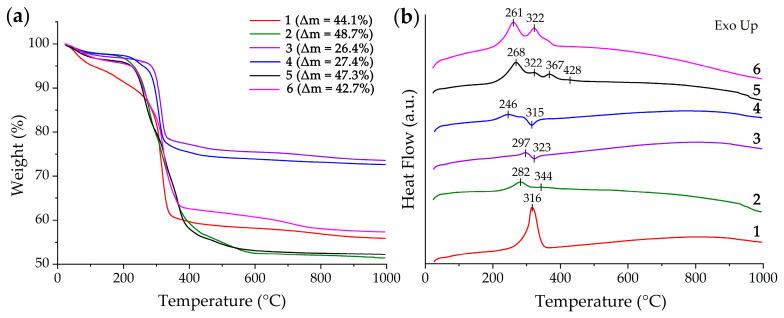
TGA (**a**) and DSC (**b**) curves of the obtained semi-products after drying (100 °C, 3 h).

To study the behavior of the obtained semi-products in the temperature range of 25–1000 °C, a synchronous thermal analysis was applied (Figure 1). As can be seen from the thermograms, the powders (Figure 1a) can be divided into 4 groups according to the nature of their thermal transformation and the resulting mass loss: group 1 (samples 2 and 5), group 2 (sample 1), group 3 (sample 6), and group 4 (samples 3 and 4). This grouping of semi-products agrees well with their synthesis conditions. Thus, group 1 powders corresponded to the maximum concentration (0.100 mol/L) of nickel chloride in the reaction system during synthesis. The maximum ratio of triethanolamine and nickel chloride concentrations (c(TEA)/c(Ni^2+^) = 80) was observed for group 2 (sample 1). In the case of group 3 (sample 6), this ratio (c(TEA)/c(Ni^2+^) = 16) is significantly lower than in group 2 but at the same time greatly exceeds this parameter with respect to other groups. Group 2 semi-products differ from the others in the lowest triethanolamine concentration in the reaction systems (0.010 and 0.050 for samples 3 and 4, respectively). The above grouping of the samples generally agrees fairly well with the type of DSC curves as well (Figure 1b). Thus, at the first stage of heating (in the interval 25–200 °C) for all samples (except for samples 1 and 3), there is a 1.5–3% weight loss associated with the evaporation of the residual solvent and sorbed atmospheric gases. For samples 1 and 3, the first step of mass loss is more extended in temperature (25–250 °C), and the corresponding Δm values are about 12 and 3%, respectively. The second mass loss step for groups 1, 3, and 4 (except for sample 3), accompanied by exothermic effects, is in the region of 200–300 °C, and the mass loss in this case has a value of 16–18, 8, and 3%, respectively. Furthermore, as the temperature rises above 300 °C, the intensification of mass loss is observed in all samples. For groups 1 and 3, this temperature range is wider (300–400 °C; Δm value of 22–24%) and energy release is observed, whereas in the case of groups 2 and 4, this interval is considerably narrower (300–350 °C, Δm (group 2) = 27%; Δm (group 4) ~ 17%). The mass loss for group 2 is accompanied by an intense exo-effect, whereas the samples of group 4 demonstrate energy absorption that is essentially different from other powders (in particular, the presence of similar endo-effects that may refer to the decomposition of β-Ni(OH)_2_ to NiO [67]). Such features testify to significant differences in the chemical composition or crystalline structure of the above semi-products. A further increase in temperature leads to a considerable slowing of the mass loss for the samples of groups 2 and 4. For group 1 samples, an ~8% mass loss step is observed in the range of 400–600 °C. For group 3, a step of mass loss (~5%) is visible enough when using a wider temperature range (400–750 °C). The optimal conditions for the additional heat treatment of semi-products (400 °C, 1 h) and for their complete decomposition and nickel (II) oxide crystallization were determined by the analysis results. Thus, the synchronous thermal analysis data for the obtained semi-products indicate a significant influence of the synthesis conditions on their thermal transformation, and probably had an influence on their chemical composition and crystal structure as well.

**Figure 2:**
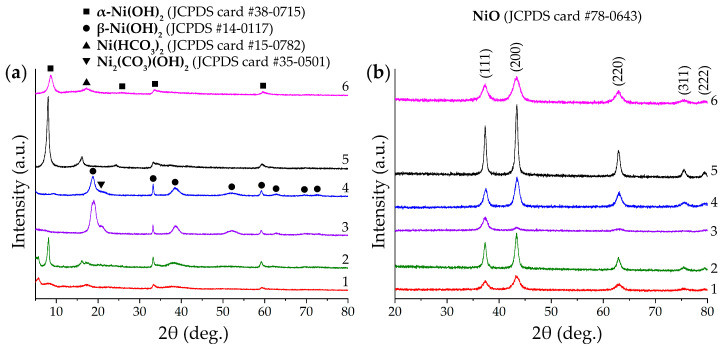
XRD patterns of semi-products (**a**) and corresponding NiO powders (**b**).

An X-ray diffraction analysis of the semi-products (Figure 2a) made it possible to determine the significant dependence of the obtained powders’ crystal structures on their synthesis conditions. The X-ray diffraction patterns show that the main component of samples 1 and 2 is α-Ni(OH)_2_ (JCPDS card #38–0715), and the presence of low-angle basal reflections indicates the layered nature of their crystal structure [68]. In the case of sample 1, there is a reduced crystallinity degree, which may be due to the intercalation of the anions present in the reaction system into the material structure, which leads to the stacking rearrangement of the layered hydroxide unit cell and the increased degree of disorder in the stacked structure. The formation of carbonate ions in the reaction systems during hydrothermal treatment suggests that samples 1 and 2 have the composition of [Ni(OH)_2–*x*_(CO_3_)_0.5x_·*y*H_2_O] (where *x* = 0.2–0.4, *y* = 0.6–1.0) (JCPDS card #22–752) [69]. For samples 5 and 6, the main component also has a structure similar to the α-modification of nickel (II) hydroxide. In this case (in contrast to samples 1 and 2), there are no reflexes below 7° of the 2θ angle in the XRD patterns, although a general shift of the (003) plane to a low angle region is also observed, indicating the formation of a layered structure of the material. The reflections in the 2θ range of 16–17° on the XRD patterns of the samples 1, 2, 5, and 6 could be attributed to the admixture of the nickel hydrocarbonate of the composition Ni(HCO_3_)_2_ (JCPDS card #15–0782) [70]. Samples 3 and 4 significantly differ from the others in terms of their crystal structure. As can be seen, β-Ni(OH)_2_ (JCPDS #14–0117) [71] is the major component for these semi-products, and the corresponding major diffraction reflections are located around 18.7°, 33.2°, 38.6°, 52.3°, 59.1°, and 62.8°. It should be noted that at 2θ ~ 20.8°, an additional diffraction peak is also observed, which may be related to the presence of Ni_2_(CO_3_)(OH)_2_ (JCPDS card #35–0501) [70]. Thus, the X-ray diffraction analysis of the semi-products suggests that by reducing the triethanolamine concentration in the reaction system to 0.010–0.050 mol/L with a simultaneous decrease of the c(TEA)/c(Ni^2+^) ratio to 2, there is a tendency for the formation of β-Ni(OH)_2_-based powders. A significant increase in the triethanolamine concentration (up to 0.400 mol/L) at a low nickel chloride concentration (0.005 mol/L) leads to the formation of layered nickel (II) hydroxide in α-modification.

Additional heat treatment of the semi-products at 400 °C for 1 h, as can be seen from the corresponding XRD patterns (Figure 2b), leads to their complete decomposition and leads to the formation of the cubic NiO crystal structure (JCPDS card #78–0643, space group Fm-3 m) [72]. Using the obtained data in accordance with the Debye–Scherrer formula, the average size of the coherent scattering region (CSR) for NiO powders was estimated. As a result, it was determined that this parameter value is consistent with the hydrothermal synthesis conditions and thermal analysis data. Thus, the minimum values of CSR corresponded to samples 1 (8 ± 1 nm) and 6 (6 ± 1 nm), for which the c(TEA)/c(Ni^2+^) ratio was the highest at the stage of intermediates synthesis and the resulting mass loss on the TGA curves had an average value. For samples 2 and 5, the CSR values were the maximum (sample 2: 14 ± 1 nm, sample 5: 19 ± 2 nm), which agrees with the maximum concentration of nickel chloride in the reaction systems (0.100 mol/L) and highest Δm values from the thermal analysis. The group of samples 3 and 4 has an intermediate CSR value (sample 3: 9 ± 1 nm, sample 4: 10 ± 1 nm); this difference also corresponds well with the synthesis conditions of the intermediates (the minimum triethanolamine concentration at the lowest values of the c(TEA)/c(Ni^2+^)) ratio, their thermal analysis results (the minimum resulting mass loss), and the crystal structure features (on β-Ni(OH)_2_-based powders).

**Figure 3:**
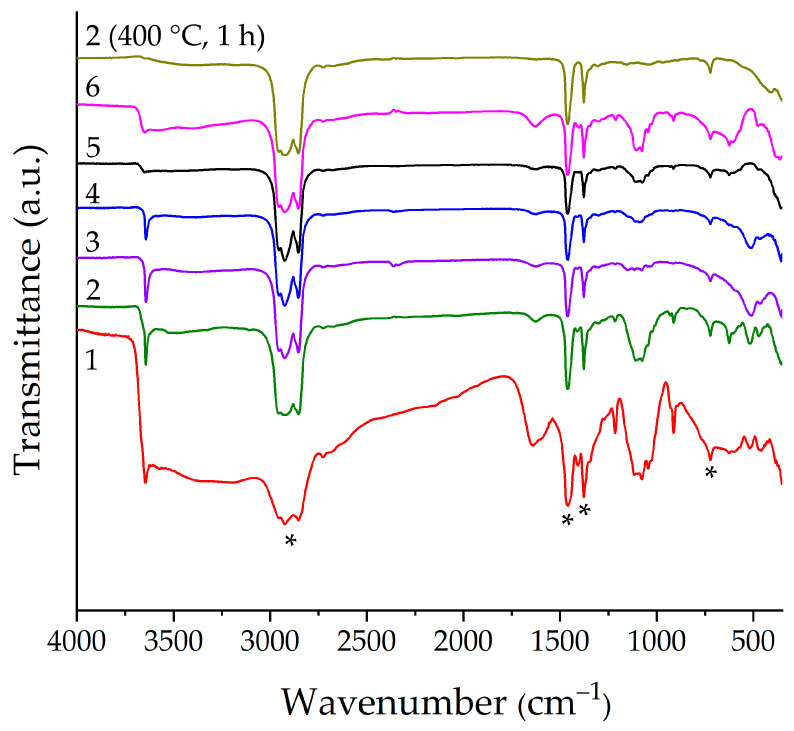
IR spectra of the semi-products (samples 1–6) after drying (100 °C, 3 h) and NiO powder (sample 2, as an example) obtained via additional heat treatment at 400 °C for 1 h; the “*” marker indicates Vaseline oil bands.

The determination of functional groups in the obtained semi-products was carried out using FTIR spectroscopy (Figure 3). As can be seen from the spectra for samples 1, 2, 3, and 4, a narrow absorption band with a maximum of about 3650 cm^−1^ is clearly observed, which is related to the stretching vibration of free hydroxyl groups of nickel hydroxide [73,74]. For samples 5 and 6, this band is less pronounced. A broad absorption band in the 3100–3750 cm^−1^ region is observed for all intermediates and refers to the stretching vibrations of the bound OH groups. It should be noted that these absorption bands are the most intense for sample 1. For all samples, there is also an absorption band with a maximum of about 1630 cm^−1^, which refers to the bending vibrations of the OH groups. The spectra of the studied semi-products also show that for all samples there is a complex-form absorption band at 950–1240 cm^−1^ as well as a narrow band around 912 cm^−1^ (for samples 1, 2, 5, and 6) related to carbonate ions vibrations [75], which is in good agreement with the XRD results concerning the presence of Ni(HCO_3_)_2_ in samples 1, 2, 5, and 6 and Ni_2_(CO_3_)(OH)_2_ in samples 3 and 4. In addition, for samples 3 and 4, these absorption bands are characterized by a lower intensity, which is probably due to the lower carbonate ions content in the above impurity composition and is manifested by a lower Δm value on the corresponding TGA curves. After additional heat treatment of semi-products at 400 °C, there is a complete disappearance of absorption bands related to the functional groups considered above. Thus, the spectrum of NiO powder obtained using sample 2 is provided as an example. In the corresponding spectrum, in addition to the Vaseline oil absorption bands, there is only one band with a maximum at 420 cm^−1^ that is associated with the Ni–O group stretching vibrations [76].

**Figure 4:**
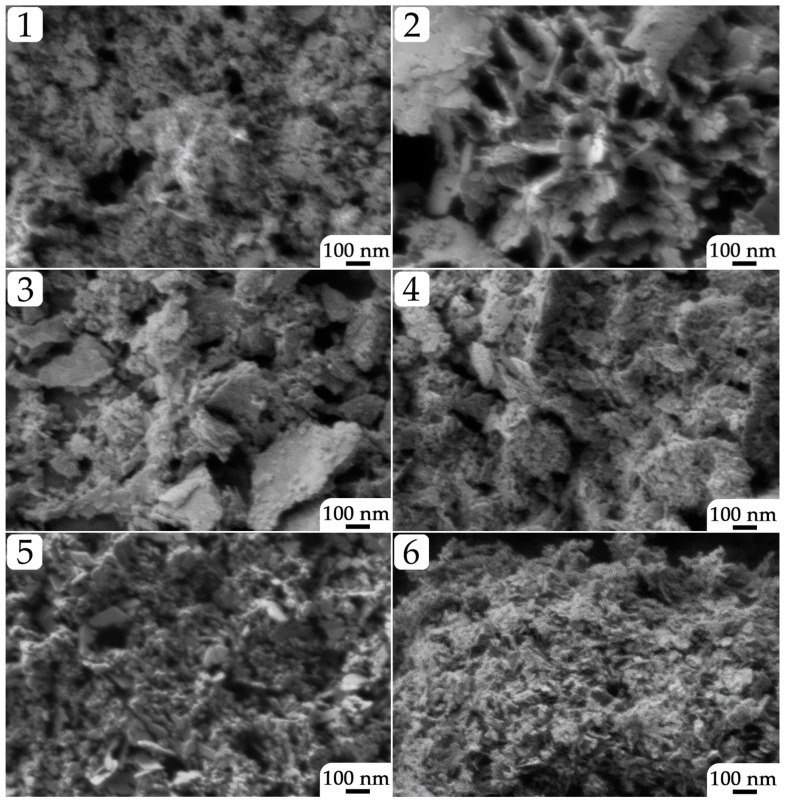
Microstructure of the NiO nanopowders (samples 1–6) obtained after additional heat treatment (400 °C, 1 h; according to SEM data).

The NiO powders microstructure after additional heat treatment at 400 °C was studied using scanning electron microscopy (SEM; Figure 4). It can be seen that the obtained powders are agglomerates consisting of differently shaped particles. Thus, in the case of samples 1, 5, and 6 synthesized at a higher triethanolamine concentration (0.400 mol/L), it was found that plate-like structures of slightly distorted shape are present on the surface of the agglomerates. Decreasing the precipitant concentration while maintaining a high nickel chloride concentration (sample 2) results in the formation of a hierarchically organized material consisting of flat, somewhat elongated particles (average size about 50 nm), which form nanosheets that are about 12 nm thick and are ordered at an angle to one another in the form of honeycomb agglomerates. A further decrease in the reagents concentration while maintaining the c(TEA)/c(Ni^2+^) ratio at 2 probably leads to the degradation of the self-organized structure and formation of individual planar agglomerates whose average size and thickness increase from 150 (sample 4) to 300 nm (sample 3) and from 12 (sample 4) to 45 nm (sample 3), respectively. At the same time, the surface of the observed agglomerates within these two samples are covered by smaller particles whose average size increases from 13 (sample 4) to 25 nm (sample 3).

**Figure 5:**
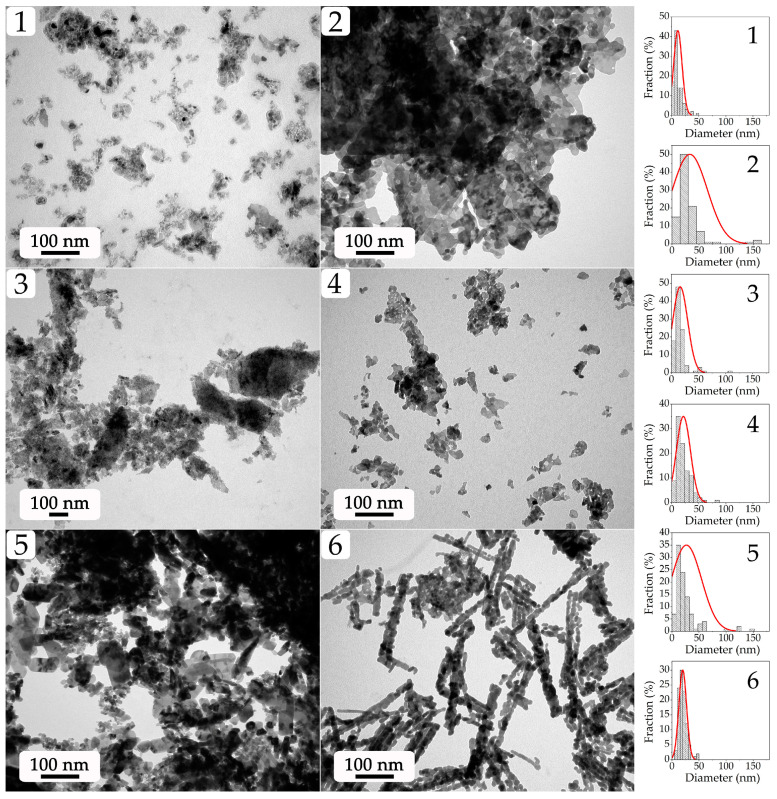
Microstructure of the obtained NiO nanopowders (samples 1–6; according to the TEM data) and the corresponding particle size distributions (right column).

Transmission electron microscopy (TEM; Figure 5) allowed for a more detailed study of the microstructure features of the obtained oxide nanopowders. The TEM results are generally consistent with the SEM data. Thus, sample 1 obtained in the case of a large excess of the precipitant mainly consists of small particles that are about 10 nm in size and nanosheet fragments with small thicknesses. With a decrease in the nickel chloride concentration in the reaction system while retaining a large amount of triethanolamine (sample 5), a polymodal particle size distribution is observed; there are highly dispersed particles with an average size of about 8 nm, larger faceted particles (about 45 nm), and slightly elongated plate-like structures (about 100 nm long) as observed earlier in the SEM images. In the case of sample 6, the use of TEM allowed for the specification of planar particles observed in the corresponding microphotograph (Figure 4), which apparently consisted of rod-like structures (about 230 nm long) and in turn are organized from smaller particles with a size of about 14 nm. Sample 2, for which SEM data previously indicated a hierarchical organization of the microstructure, according to TEM results consists of lamellar structures (nanosheets) with clear edges on the surface, where small particles of about 15 nm can be observed. In the case of samples 3 and 4, the micrographs show fragments of planar agglomerates consisting of small nanosheets with an average size of 30 (sample 4) to 40 (sample 3) nm, along with highly dispersed particles ranging from 13 (sample 4) to 10 (sample 3) nm. The corresponding histograms clearly show the bimodal particle size distribution for samples 2 and 5 obtained from reaction systems with the highest nickel chloride concentration. As can be seen, the microstructural features (in particular, dispersity) of the obtained NiO powders agree quite well with the CSR values determined by the XRD data.

Based on the microstructure analysis of the obtained NiO nanopowders, to further form the functional ink and subsequent microextrusion printing of the electrode coating of the composition NiO— (CeO_2_)_0.80_(Sm_2_O_3_)_0.20_ (NiO—SDC), sample 2 was chosen, which is characterized by the most ordered hierarchically organized morphology.

### 2.2. Characterization of the Printed NiO—SDC Film

**Figure 6:**
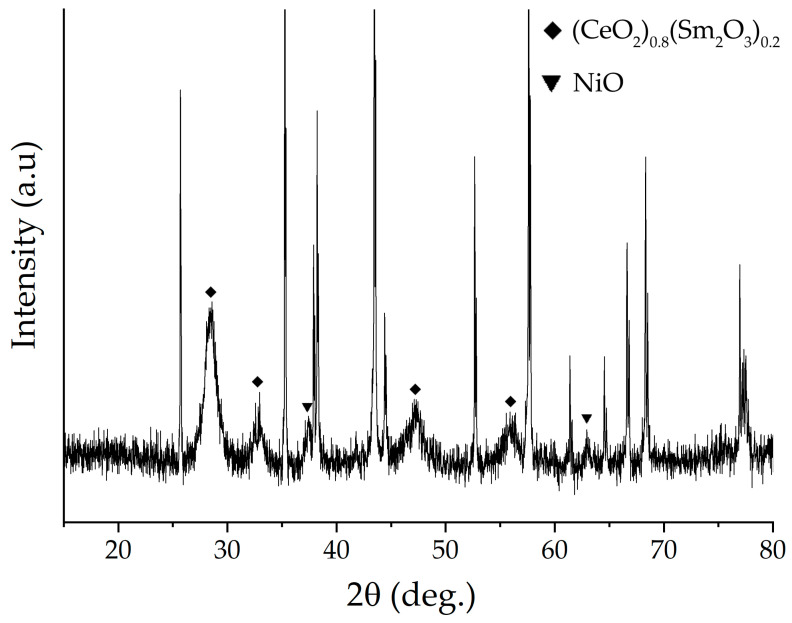
XRD pattern of the NiO–SDC composite film printed on an Al_2_O_3_ substrate surface.

The crystal structure of the printed NiO—SDC composite film was studied using an X-ray diffraction analysis (Figure 6). The corresponding XRD patterns demonstrate that in addition to the intense narrow reflexes related to the substrate, signals from the film components ((CeO_2_)_0.80_(Sm_2_O_3_)_0.20_ and NiO) are present as well. The low-intensity character of the reflexes from the investigated planar structure also indirectly confirms its small thickness, and the broadened reflexes testify to the preservation of the high dispersion of components (in particular, for the SDC phase, the average size of the CSR is 8 nm, and the signals from NiO particles largely overlap the substrate background, which complicates the estimation of this parameter). Thus, the XRD analysis of the obtained film surface confirmed its composite structure, the preservation of the crystal lattice type of its constituents, and the absence of impurities from the possible interaction with the substrate material.

**Figure 7:**
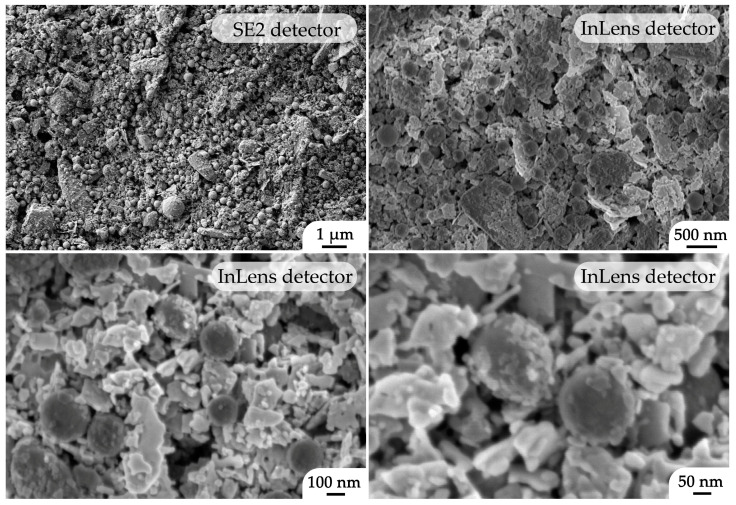
Microstructure of the NiO–SDC composite film printed on an Al_2_O_3_ substrate surface (according to SEM data).

The resulting film microstructure was analyzed using SEM (Figure 7). Thus, SE2 and InLens secondary electron detectors were employed to analyze the material topography and component dispersion, respectively. As can be observed from the micrographs, the film consists of uniformly distributed nanospheres (about 250 nm in diameter) related to samarium-doped ceria, as well as nanosheets and smaller particles of nickel (II) oxide. At the same time, (CeO_2_)_0.80_(Sm_2_O_3_)_0.20_ nanospheres are covered by a thin shell of NiO particles ranging 20–30 nm, which probably formed during the functional ink preparation by means of components self-organization. It should also be noted that the character of their microstructure in the process of ink preparation, film printing, and additional heat treatment was preserved and corresponds to the used nanopowders. In addition, the formed NiO—SDC composite film has a sufficiently high porosity, which is an important parameter for the anode components to ensure their efficiency. The study of the material surface also confirmed the absence of any impurities that differ from the main components in terms of dispersity or particle shape.

**Figure 8:**
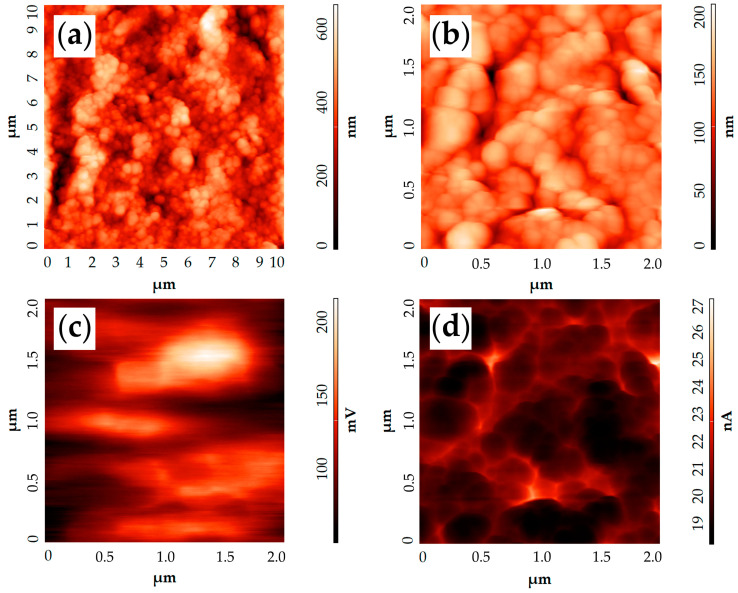
AFM results of the NiO—SDC film under study: (**a**,**b**)—topography; (**c**)—surface potential distribution map; (**d**)—capacitance gradient map of the “probe tip-sample microregion” capacitor.

The AFM results (Figure 8) for the investigated composite film agree well with the SEM data. From the topographic images (Figure 8a,b), it was determined that the surface of the material has an arithmetic mean profile deviation (R_a_) of about 88 nm and consists of nanoparticles of different shapes and sizes. In particular, spherical particles with diameters in the range of 170–220 nm are clearly visible along with particles of more elongated shape. The length of these particles is mostly close to the diameter of the spheres, although submicron-size formations (up to 600 nm) are also found. From the analysis of the microstructure of powders used for the functional ink preparation, it follows that the nanospheres belong to the oxide composition (CeO_2_)_0.80_(Sm_2_O_3_)_0.20_, while the elongated particles belong to the nickel oxide nanosheets. The different chemical nature of these particles is confirmed by the KPFM data (Figure 8c). The resulting surface potential distribution map shows that nanospheres correspond to darker areas, while the more elongated formations correspond to lighter areas. The difference in the potential values in this case reaches 150 mV and, overall, the contrast between the different potentials of the film sections is visible enough on the map. At the same time, the capacitance gradient map of the capacitor “probe tip-sample microregion” (Figure 8d), obtained by semi-contact SCM, does not show a significant contrast-probably, it mainly reflects the depleted zones at the boundaries between particles.

The work function value of the material surface was determined based on the KPFM results. For samarium-doped ceria nanospheres, the work function value was 4.61 eV, and for NiO particles it was 4.5 eV. This parameter of (CeO_2_)_0.80_(Sm_2_O_3_)_0.20_ oxide is slightly higher than the value (4.44 eV) we obtained for the material of the same composition synthesized under different conditions in our previous research [64]. This, as well as the microstructural differences, is presumably explained by the peculiarities of the synthesis conditions. Probably, the material formed under these conditions is characterized by a lower concentration of defects (primarily oxygen vacancies), which results in an increased work function value. The work function for NiO also differs from the previously obtained value for a similar hydrothermal synthesis product in the presence of triethanolamine (but at a lower temperature); in Ref. [77], we obtained a value of 4.75 eV. NiO is known to be a p-type semiconductor because of the cationic vacancies it contains [78]. Such vacancies can be considered as “acceptors” of electrons and, as a consequence, as their concentration increases, the Fermi level decreases and the work function value grows. Accordingly, it can be assumed that the higher-temperature synthesis in our case promotes the formation of a more stoichiometric NiO oxide, which leads to a lower work function value. The formed composite film thickness was also determined by the AFM analysis, where it was determined to be 5 μm.

**Figure 9:**
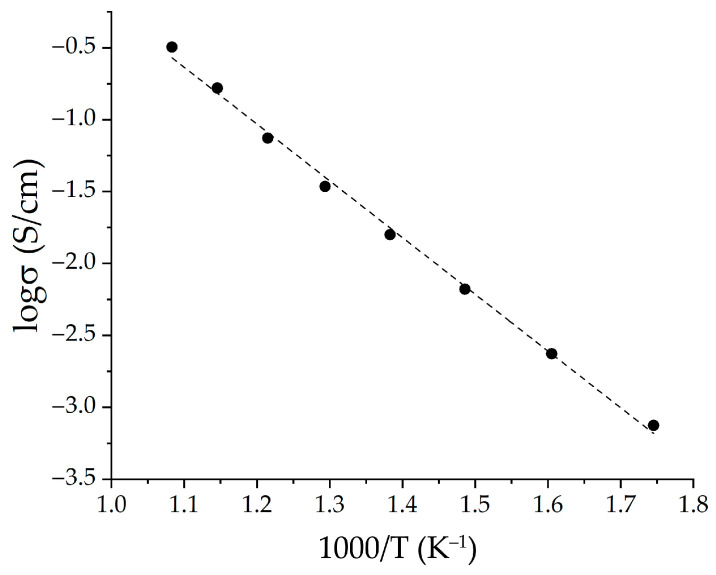
Arrhenius plot for NiO—SDC coating in the temperature range of 300–650 °C (the black markers indicate experimental values, and the dashed line is the result of linear approximation).

The conductivity of the NiO—SDC composite film was evaluated using four-probe DC measurements in the temperature range of 300–650 °C. It is known that in the case of such materials, both components influence the final conductivity value, with the nickel oxide determining the electronic conductivity and the cerium dioxide-based solid solution influencing the ionic conductivity [79]. It can be seen (Figure 9) that the obtained dependence is linear in the entirety of the range under consideration, and the conductivity value increases by several orders of magnitude (from 7.5–10^−4^ to 0.3 S/cm). These conductivity values generally correspond to those obtained for similar anode materials; however, they can be increased by an additional high-temperature treatment [79,80]. The activation energy of conductivity was calculated using the following formula:

(1) $$\sigma =A/T\cdot e^{\frac{-Ea}{kT}},$$

where *A* is the pre-exponential factor, *T* is the absolute temperature, *k* is the Boltzmann constant, and *Ea* is the activation energy of conductivity.

In our case, the activation energy for the 500–650 °C region, which is of the greatest interest in the context of the intermediate-temperature SOFCs operating temperatures, was 0.42 eV, which also agrees with the data for NiO—SDC composites [81].

## 3. Materials and Methods

### 3.1. Materials

NiCl_2_·6H_2_O (>98%, RUSHIM, Moscow, Russia), (triethanolamine (C_6_H_15_NO_3_, 99%, Chimmed, Moscow, Russia), α-terpineol (>97%, Vekton, St. Petersburg, Russia), and ethylcellulose (48.0–49.5% (*w*/*w*) ethoxyl basis, Sigma Aldrich, St. Louis, MO, USA) were used in this work without further purification.

### 3.2. Hydrothermal Synthesis of NiO Nanopowders

The formation of NiO nanopowders was carried out using the hydrothermal method. Thus, at the first stage, the reaction systems representing 40 mL of aqueous solutions with different concentration ratios of nickel chloride and triethanolamine (Table 1) were prepared and subjected to hydrothermal treatment in a 100 mL Teflon-lined stainless-steel autoclave at 200 °C for 1 h (heating rate of 1.5 °C/min). After the natural cooling of the systems, the solid phase particles that formed during the synthesis were separated from the mother liquors and washed with distilled water using a cyclic centrifugation technique; the semi-products that were obtained were further dried (100 °C, 3 h) and additionally heat-treated (400 °C, 1 h) for their decomposition and nickel (II) oxide crystallization.

### 3.3. Microextrusion Printing of NiO—SDC Electrode Film

**Figure 10:**
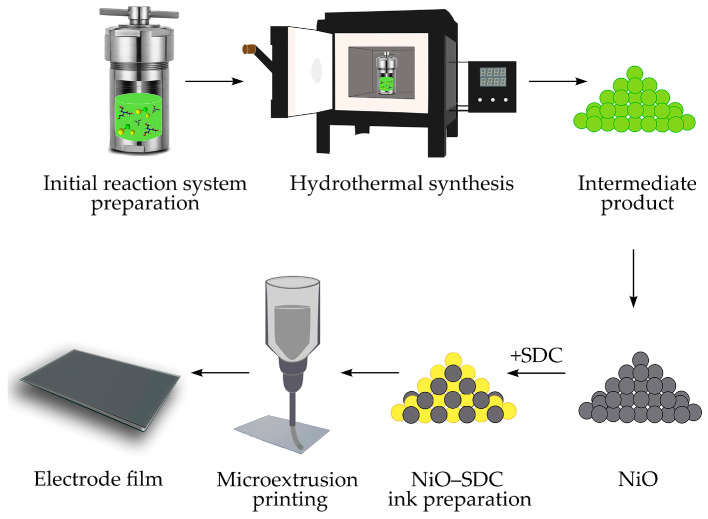
Schematic illustration of NiO synthesis and microplotter printing of the corresponding NiO—SDC film on an Al_2_O_3_ substrate.

In order to further develop the NiO—(CeO_2_)_0.80_(Sm_2_O_3_)_0.20_ composite electrode film, the corresponding functional inks were prepared based on the obtained nanopowder of nickel oxide (sample 2) and previously synthesized oxide of (CeO_2_)_0.80_(Sm_2_O_3_)_0.20_ composition [82] in a 50:50 wt.% ratio, to which α-terpineol was added as a solvent (ethylcellulose, 20 wt.% was used as a binder). The total mass fraction of oxide particles in the composition of the obtained composite functional ink was 15%. The features of the microextrusion printing technique used for oxide films fabrication were described in our previous works [63,64]. In the current study, the NiO—SDC film (lateral dimensions 6 × 8 mm) was deposited on the surface of a polycrystalline Al_2_O_3_ substrate (dimensions 0.5 × 6 × 8 mm, R_a_ = 100 nm). After the printing process was completed, a step-drying of the material was conducted in the temperature range of 40–60 °C (5 h), and additional heat treatment (400 °C, 1 h) was performed to remove residual solvent and decompose the organic components in the functional ink. For electrophysical measurements, four parallel Ag strip electrodes were also applied to the surface of the formed composite film using microextrusion printing. The technological stages of NiO nanopowders formation and the corresponding coating printing are shown in Figure 10.

### 3.4. Instrumentation

The semi-products obtained during the hydrothermal synthesis and subsequent drying were characterized using a simultaneous thermal analysis (TGA/DSC) with an SDT Q-600 thermal analyzer (TA Instruments, New Castle, DE, USA). Controlled heating was carried out in the temperature range of 25–1000 °C at a speed of 10 °/min, and the air flow rate was 250 mL/min. The samples weight was varied in the range of 10–15 mg.

The Fourier-transform infrared transmission spectra of the powders under study were recorded on an InfraLUM FT-08 FTIR spectrometer (Lumex, St. Petersburg, Russia) in the wavenumber interval of 350–4000 cm^−1^ (signal accumulation time 15 s, resolution 1 cm^−1^). For this purpose, suspensions of the corresponding samples were prepared in Vaseline oil and placed between two potassium bromide glasses.

The crystal structure of the powders and composite film were studied using an X-ray diffraction analysis on a Bruker D8 Advance diffractometer (CuKα radiation, *λ* = 1.5418 Å, Ni-filter, E = 40 keV, I = 40 mA, 2θ angle range 5–80°, resolution—0.02°, signal accumulation time at a single point was 0.3 s). To estimate the average CSR size of NiO powders, the Debye–Scherrer equation was used:

(2) $$D=\frac{k\times \lambda }{\beta \times cos\theta },$$

where *D*, *λ*, *k*, *β*, and *θ* are the CSR size, X-ray wavelength, Scherrer’s constant, full width at half maximum, and diffraction angle, respectively.

The microstructure features of the powders were studied using scanning electron microscopy (Carl Zeiss NVision-40, Carl Zeiss, Inc., Oberkochen, Germany) and transmission electron microscopy (JEOL JEM-1011, JEOL Ltd., Akishima, Tokyo, Japan). The microstructure character of the resulting composite film was also studied using SEM. Additional analysis of the morphology and local electrophysical properties of the printed NiO—SDC film was performed using atomic force microscopy. For this purpose, a Solver Pro-M scanning probe microscope (NT-MDT, Zelenograd, Russia) and ETALON HA-HR probes (ScanSens, Bremen, Germany) with tungsten carbide-based conductive coating (resonance frequency ~213 kHz, tip rounding radius <35 nm) were used in semicontact AFM, Kelvin probe force microscopy, and scanning capacitance microscopy modes. All measurements were performed in air. The electronic work functions of different sections of the material were determined by subtracting the contact potential between the probe and the film from the previously known work function value of the probe tip (4.68 eV).

The electrical conductivity of the printed anode composite film was measured using a four-probe method on a direct current in the temperature range of 300–650 °C in air using a P-45X potentiostat/galvanostat (Electrochemical Instruments, Chernogolovka, Russia). The total conductivity of the sample was calculated by the formula:

(3) $$\sigma =\frac{I\times L}{U\times S},$$

where *I* is the current flowing through the sample (A); *L* is the distance between the two internal electrodes (cm); *U* is the voltage between the two internal electrodes (V); and *S* is the cross-sectional area of the test material (cm^2^).

## 4. Conclusions

The hydrothermal synthesis of nickel oxide in the presence of triethanolamine was studied, and the dependencies between the synthesis conditions, thermal behavior, crystal structure features, phase composition, and microstructure of the semi-products and target oxide nanopowders were established. It was shown that upon a decrease in the triethanolamine concentration in the reaction system down to 0.010–0.050 mol/L and a simultaneous decrease in the c(TEA)/c(Ni^2+^) ratio to 2, a formation of β-Ni(OH)_2_-based powders tends to occur. A significant increase in the triethanolamine concentration (up to 0.400 mol/L) at a low-nickel chloride concentration (0.005 mol/L) leads to the formation of layered nickel (II) hydroxide in α-modification. For intermediates whose main component is α-Ni(OH)_2_, nickel hydrocarbonate having the Ni(HCO_3_)_2_ composition was found to be a crystalline impurity. In the case where the main component is β-Ni(OH)_2_, it is accompanied by a Ni_2_(CO_3_)(OH)_2_ impurity. It was determined that an additional heat treatment of the semi-products at 400 °C for 1 h leads to their complete decomposition and formation of a cubic NiO crystal structure. It was found that the minimum values of the CSR size correspond to the NiO powders (6–8 nm) for the stage of semi-product synthesis at which the c(TEA)/c(Ni^2+^) ratios were the highest and the final mass loss on the TGA curves had an average value. For the samples obtained at the maximum nickel chloride concentration in the reaction systems (0.100 mol/L), the CSR values were maximum (14–19 nm), which was also evident in the highest Δm values from the thermal analysis results. Using one of the obtained NiO nanopowders and the previously synthesized nanospheres of the (CeO_2_)_0.80_(Sm_2_O_3_)_0.20_ composition, functional ink was obtained, and a NiO—SDC composite anode film was formed by using microextrusion printing. It was demonstrated that the crystal structure, dispersity, and microstructure character of the obtained material correspond to the initial nanopowders. It was also determined that the material has a sufficiently high porosity, which is an important parameter for the anode components in order to ensure their efficiency. Using the AFM analysis, it was found that the film thickness is 5 μm. The local electrophysical properties of the composite film were studied using KPFM and SCM techniques. In addition, the value of its conductivity was evaluated by the four-probe method with a direct current and in the temperature range of 300–650 °C. As a result, it was found that the obtained temperature dependence is linear in the entirety of the considered range, and the conductivity value increases by several orders of magnitude (from 7.5–10^−4^ to 0.3 S/cm). The activation energy for the 500–650 °C region, which is of the greatest interest in the context of intermediate-temperature SOFCs operating temperatures, was 0.42 eV, which also agrees with the data for NiO—SDC composites.

## Figures and Tables

**Table 1:** Designation of samples and reagent concentrations in the corresponding reaction systems.

Sample	c(Ni^2+^), mol/L	c(TEA), mol/L	c(TEA)/c(Ni^2+^)
1	0.005	0.400	80
2	0.100	0.200	2
3	0.005	0.010	2
4	0.025	0.050	2
5	0.100	0.400	4
6	0.025	0.400	16

## Data Availability

The data presented in this study are available in this article.
